# A case of persistent left superior vena cava with absent right superior vena cava draining into dilated coronary sinus: magnetic resonance imaging and computed tomography findings

**DOI:** 10.1186/1749-8090-8-S1-O67

**Published:** 2013-09-11

**Authors:** T Batinić, Z Jurišić, I Štula

**Affiliations:** 1 Department of Radiology, University Hospital Split, Split, Croatia; 2 Department of Cardiology, University Hospital Split, Split, Croatia

## Background

We report a case of a persistent left superior vena cava (PLSVC) with absent right superior vena cava (RSVC). It is a very rare congenital anomaly also known as isolated PLSVC.

This venous malformation was identified in a 75-year-old woman during cardiac magnetic resonance imaging (MRI), which was performed with the suspicion of a paracardiac mass.

## Methods

We performed an MRI and a multislice computed tomography (MSCT) evaluation.

## Results

Cardiac MRI revealed a persistent left superior vena cava which descended on the left side of the mediastinum and drained into the right atrium (RA) via a markedly dilated coronary sinus (CS) which mimicked a paracardiac mass. The RSVC was absent.

These findings were confirmed by MR and MSCT venography.

The patient had no additional cardiac abnormality.

## Conclusions

Although PLSVC is usually asymptomatic, it is important to be aware of its existence, since it may cause problems performing central venous catheterization, pacemaker implantation and cardiothoracic surgery.

This anomaly is also associated with high incidence of congenital heart disease, arrhytmias and conduction disturbances.

Modern imaging techniques including computed tomography and magnetic resonance imaging provide precise diagnosis of this anomaly.

